# The Modern Plant Breeding Triangle: Optimizing the Use of Genomics, Phenomics, and Enviromics Data

**DOI:** 10.3389/fpls.2021.651480

**Published:** 2021-04-16

**Authors:** Jose Crossa, Roberto Fritsche-Neto, Osval A. Montesinos-Lopez, Germano Costa-Neto, Susanne Dreisigacker, Abelardo Montesinos-Lopez, Alison R. Bentley

**Affiliations:** ^1^International Maize and Wheat Improvement Center (CIMMYT), Carretera México-Veracruz, de Mexico, Mexico; ^2^Colegio de Postgraduados, Montecillo, Edo. de Mexico, Mexico; ^3^Department of Genetics, “Luiz de Queiroz” Agriculture College, University of São Paulo, São Paulo, Brazil; ^4^Facultad de Telemática, Universidad de Colima, Colima, Mexico; ^5^Departamento de Matemáticas, Centro Universitario de Ciencias Exactas e Ingenierías (CUCEI), Universidad de Guadalajara, Guadalajara, Mexico

**Keywords:** genomics, phenomics, enviromics, high throughput phenotype, multi-trait, multi-environment

## Introduction

Continued increases in genetic gain demonstrate the success of established public and private plant breeding programs. Nevertheless, in the last two decades, a growing body of modern technologies has been developed and now awaits efficient integration into traditional breeding pipelines. This integration offers attractive benefits, yet comes with the challenges of making modifications in established and operational systems, a recent example of which is rice breeding (Collard et al., 2019). Newly available technologies, genomics rapid cycling (Crossa et al., 2017), high throughput phenotyping (HTP, phenomics) (Montesinos-López et al., 2017) and historical descriptions of environmental relatedness (enviromics) (Costa-Neto et al., 2020a,b; Resende et al., 2020; Rogers et al., 2021) are crucial to improving conventional breeding schemes and increasing genetic gain. Integrating these new technologies into routine breeding pipelines will support the delivery of cultivars with robust yields in the face of the expected unfavorable future environmental conditions caused by climate change and the consequently increased occurrence of biotic and abiotic stresses. Here, we briefly describe the use of these technologies and their implementation to provide cost-effective and time-saving approaches to plant breeding. We also give an overview of the interconnections between these techniques. Finally, we envision future perspectives to implement a more interconnected breeding approach that takes advantage of the so-called modern plant breeding triangle: integrating genomics, phenomics, and enviromics.

### Why Genomics for Improving Breeding?

One of the most popular uses of genomics in breeding is the prediction of breeding values. Genomic selection (GS) reduces cycle time, increases the accuracy of estimated breeding values and improves selection accuracy. For instance, in maize, the effectiveness of GS has been proven for the case of bi-parental populations (Massman et al., 2013; Beyene et al., 2015; Vivek et al., 2017), as well as in multi-parental populations (Zhang et al., 2017). Its use has also been documented in species with long generation times such as trees (Grattapaglia et al., 2018) and dairy cattle breeding, where the reduction of the breeding cycle has increased the response to selection in comparison with the progeny testing system (García-Ruiz et al., 2016).

Genomic selection has been implemented in many crops, including wheat, chickpea, cassava and rice (Roorkiwal et al., 2016; Crossa et al., 2017; Wolfe et al., 2017; Huang et al., 2019), and the number of programs that are moving from “conventional” to GS is growing. Results in wheat show that genomic predictions used early in the breeding cycle led to a substantial increase in performance in later generations (Bonnett et al., 2021 this issue).

#### Defining Foundational Core Parents for Genomic Selection-Assisted Breeding

In genomic selection, the optimization of the training set composition is an important topic because training and testing sets should be genetically related in such a way that the genetic diversity present in the testing set could be covered and captured by the diversity in the training set. Breeding programs must start forming initial foundational core parents (training populations) that represent the genetic diversity found in the current progeny and conform to the testing population(s) to the greatest extent possible (Hickey et al., 2012). These foundational parents should be extensively phenotyped in different target populations of environments and genotyped with high-density marker systems. These training sets of foundation parents will be able to produce a model with a high accuracy for current highly selected progenies (Zhang et al., 2017).

### Why Detailed Phenomics and the Use of Multi-Trait Analysis to Improve Breeding?

The most important limitation to determining accurate phenotypes has been the time and cost required to measure traits in the field. Field phenomics aims to study all plant phenotypes under a range of environmental conditions. Modern phenomics methods are able to use hyperspectral/multispectral cameras to provide hundreds of reflectance data points at discrete narrow bands in many environments and at many stages of crop development. Phenotyping technology can now be used to quickly and accurately obtain data on agronomic traits based on advancements in plant phenotyping technologies (Atkinson et al., 2018). Therefore, the main goal of a high-throughput phenotype (HTP) is to reduce the cost of data per plot and to increase the prediction accuracy early in the crop-growing season with the use of highly heritable secondary phenotypes, closely related to the selection phenotypes. The cost of processing HTP data can be minimized by using open-source software, such as FieldImageR (Matias et al., 2020).

There is evidence that multi-trait analyses improve prediction accuracies when the genetic and residual correlations are considered in the modeling process. New genomic models that take the multiple traits and the multiple environments into consideration, along with trait × environment, trait × genotype, and trait × genotype × environment interactions, offer a huge potential for the exploitation of correlations between different variables and for the differentiation between effects. Integrating current GBLUP multi-trait models with models that consider the environmental information with the two- and three-way interaction terms provides a powerful, unified, whole genome prediction model.

The Bayesian multi-trait and multi-environment model (BMTME) (Montesinos-López et al., 2016, 2019a) allows for general covariance matrices for traits and environments that capture the correlations among traits and environments better. This unified model could be implemented to select genotypes with traits measured in one environment and to predict in other, untested environments. It could also be applied to predict traits that are costly or difficult to measure in all environments.

It is crucial to obtain large and inter-operable phenomics datasets from field phenotyping. This should be used to characterize the foundational core parents in the different environments and incorporate them into the visual data collected in the different environments. These data, along with pedigree and genomic information, can be used to fit Bayesian linear mixed models to compute BLUPs of the genetic values of the material in the training set. Breeding programs should collect multi-trait data on the multi-environment used for foundational core parents and exploit possible correlations among traits that will eventually increase prediction accuracy. The genomics and phenomics of the multi-trait foundation core parents are essential for use alongside enviromics data.

### Why Enviromics to Improve Multi-Environment Trials for Genomics-Assisted Plant Breeding?

The phenotypic variation observed across diverse environments is a product of genetic and environmental variation. Thus, enviromics acts as a central bottleneck for the application of modern genomics-assisted prediction tools, especially for use across multiple environments. Novel approaches have integrated field trial data with DNA sequences using different sources of enviromics, such as linear and nonlinear reaction-norm models (e.g., Jarquín et al., 2014; Morais-Júnior et al., 2018; Millet et al., 2019; Monteverde et al., 2019; Costa-Neto et al., 2020a), crop growth model (CGM) outputs (Heslot et al., 2014; Rincent et al., 2017, 2019), CGM integrated with GS (Cooper et al., 2016; Messina et al., 2018; Robert et al., 2020) and historical weather records to predict cultivars in years to come (de los Campos et al., 2020).

For example, the strategy proposed by de los Campos et al. (2020) assesses genomic × environment (G × E) patterns learned from field trials and predicts the expected performance of a cultivar in an environment but also evaluates the expected distribution of a cultivar performance over other possible weather conditions, while accounting for uncertainty in model parameters. This is a new method for the analysis of multi-environment trials and can speed up the assessment of grain yield adaptability and stability.

Another recent example is the approach that can increase the resolution in multi-environment prediction for stability by taking advantage of large-scale enviromics with different kernel methods (Costa-Neto et al., 2020a). The environmental relatedness among field trials can be shaped using linear covariances (as proposed by Jarquín et al., 2014) and non-linear methods (Gaussian kernel, deep learning, and deep kernel) (Cuevas et al., 2016, 2017, 2018, 2019; Montesinos-López et al., 2018a,b, 2019b,c). The use of non-linear kernels has led to higher accuracy gains in the prediction of novel genotypes under known conditions, but mostly in the prediction of novel environment conditions (untested environments). This approach was expanded to take account of several environmental structures across different crop development stages (Costa-Neto et al., 2020b). For the latter, the authors observed an increased ability to explain G × E in terms of genotype-specific reaction norms for key environmental factors or key development stages. This increased ability to explain G × E was important to achieve higher accuracy gains in comparison with models without enviromic information.

In a recent research article, Rogers et al. (2021) emphasized the importance of incorporating high throughput environmental data into genomic prediction models in order to carry out predictions in new environments characterized with the same environmental characteristics. The author concluded that, among other factors, G × E interactions and environmental covariates should be incorporated into prediction models to improve prediction accuracy.

### Interconnection in Modern Plant Breeding

**Figure 1:**
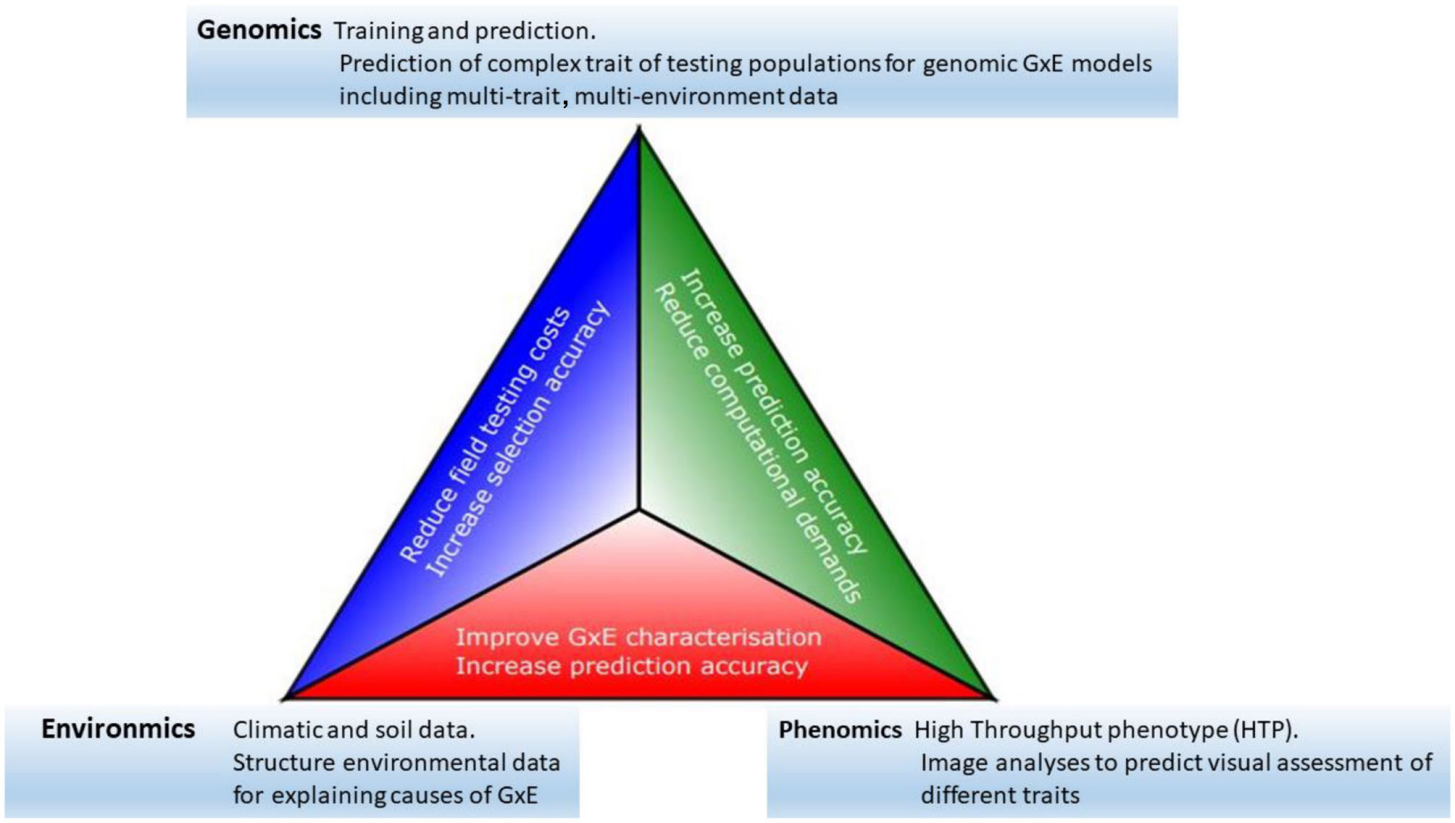
The modern plant-breeding triangle incorporates genomics, phenomics, and enviromics. Connections between each of these elements can be beneficial for the acceleration of genetic gains.

Progress toward the modernization of the statistical and quantitative genetic models for the analysis of plant breeding in multi-environment trials has become clearer as the availability of genomics, phenomics, and environments information has increased (see, among others, Vargas et al., 1998; Crossa et al., 2010; Burgueño et al., 2012; Heslot et al., 2014; Jarquín et al., 2014; Montesinos-López et al., 2017; Millet et al., 2019; Costa-Neto et al., 2020a; de los Campos et al., 2020; Robert et al., 2020). Thus, we see that all the elements described above offer a clear potential for the acceleration of genetic gains in plant breeding. However, an efficient data-based integration is required to achieve greater opportunity, particularly in terms of increasing prediction accuracy. Some of the major links between genomics, phenomics, and enviromics are outlined below, and their potential impacts are summarized in Figure 1.

### Linking Genomics and Phenomics

Linking massive data sets from genomics and phenomics has complexities that require statistical models to deal with a very large number of correlated predictors. Montesinos-López et al. (2017) proposed linking genomics and phenomics with Bayesian functional regression models that consider all available reflectance bands (250 bands or wavelength), genomic or pedigree information, the main effects of lines and environments, as well as the effects of interaction. They observed that the models with wavelength × environment interaction terms were the most accurate for the prediction of performance in three different environments and at various crop development time points. The functional regression models are parsimonious and computationally efficient because the mathematical basis functions allow the selection of only 21 beta coefficients (rather than using all 250). Recently, Lopez-Cruz et al. (2020) proposed a method to predict the genetic merit of cultivars from high-dimensional HTP data by integrating high-dimensional regressions into the standard selection index methodology.

### Linking Multi-Trait and Multi-Environment Data

Multi-trait multi-environment data (MTME) take advantage of large-scale correlations among different traits evaluated across diverse environments to train accurate GS models. Because of this, the use of GS in MTME data is a promising approach to reduce field phenotyping efforts. For example, Ibba et al. (2020) evaluated the prediction performance of 13 quality traits in wheat using two multi-trait models and five data sets based on field evaluations over two consecutive years. In the second year (testing), lines were predicted using the quality information obtained in the first year (training). For most of the quality traits, they found moderate to high prediction accuracies, suggesting that the use of GS at earlier stages could be recommendable. Overall, the results indicate that the Bayesian MTME model helps capture the correlation among traits and the correlation among years, thus increasing prediction accuracy. Finally, we envision perspectives of modeling MTME-based reaction norms involving other omics, such as phenomics and enviromics. The latter can enhance the MTME analysis in terms of creating more biological models of crop growth, development, and yield components (e.g., Robert et al., 2020).

### Interplay Genomics and Enviromics

Since the 1960s, several researchers have suggested the use of environmental information to explain the differences in cultivars due to G × E interactions (e.g., Perkins and Jinks, 1968; Freeman and Perkins, 1971; Wood, 1976; Vargas et al., 1998; Crossa et al., 1999). The use of genomics with enviromics is the basis for the prediction of cultivars across diverse growing conditions (e.g., Jarquín et al., 2014; Messina et al., 2018; Millet et al., 2019), which is useful for the prediction of global warming.

However, the efforts to implement environmental covariates into genomic selection models usually focus on a few environmental covariates such as temperature, precipitation, and sun radiation defined over specific developmental stages of the crop. With the use of large-scale envirotyping data, it is possible to design a global-scale envirotyping network of field trials to train GS models and perform “enviromic assembly” to predict a wider number of growing conditions from historical climate and soil data (R package EnvRtype, Costa-Neto et al., 2020b). In addition, research is underway for the study of model Enviromic + Genomic prediction (E-GP) to link genotype-phenotype variations, as well as to explain phenotypic variations across environments. As a predictive breeding tool, E-GP can contribute to the study of G × E structures, in which, as an exploratory tool, E-GP can contribute to the optimization of experimental networks of field trials and lead to more efficient training sets for GS (e.g., Rincent et al., 2017). In addition, for the early stages of selection, genomics and enviromics can be used to design optimized phenotyping trials and predict the breeding values of the selection candidate (Morais-Júnior et al., 2018) or single cross-hybrid prediction (Costa-Neto et al., 2020a).

Through enviromic assembly, it is possible to establish relatedness among field trials and thus use only the most representative set of experiments for training GS models. Another perspective of E-GP is the use of large-scale environmental data in training models involving genotype-specific reaction norms (e.g., Ly et al., 2018; Millet et al., 2019) and phenotypic landscapes implemented by genomics with crop growth models (CGM) (e.g., Messina et al., 2018; Bustos-Korts et al., 2019; Robert et al., 2020). The possible use of image-based responses related to main environmental stresses, such as heat and drought-stress, can also boost the implementation of genomic-assisted platforms for predictive purposes and are capable of better representing the plant-environment interplay.

### Future Perspectives

In order to meet the well-documented challenges of food and nutrition security, there is a pressing need to use new technologies to accelerate the progress of plant breeding. These methods can be incorporated into conventional phenotypic breeding programs or help redesign established phenotypic breeding pipelines to enable a gradual shift toward a more data-driven perspective. The benefits of phenomics and enviromics together in benchmark genomic pipelines offer the potential to deliver larger increases in accuracy and efficiency of breeding pipelines when we select better-adapted genotypes in a cost-effective manner, as well as in a reduced timeframe. Genomics, phenomics, multi-trait, and enviromics analyses are interconnected, and their use can be optimized based on resources and program structure. Together, they offer a pathway for conventional phenotypic breeding to envision a diverse set of opportunities to accelerate genetic gains.

## Author Contributions

JC prepared the first drafts of the opinion, RF-N, OM-L, GC-N, SD, and AM-L read and corrected the first version. AB produced several reviews of the documents and worked with JC to finalize the definitive version. All authors contributed to the article and approved the submitted version.

## Conflict of Interest

The authors declare that the research was conducted in the absence of any commercial or financial relationships that could be construed as a potential conflict of interest.
